# Effects of tumor metabolic microenvironment on regulatory T cells

**DOI:** 10.1186/s12943-018-0913-y

**Published:** 2018-11-26

**Authors:** Yi-an Wang, Xiao-Ling Li, Yong-Zhen Mo, Chun-Mei Fan, Le Tang, Fang Xiong, Can Guo, Bo Xiang, Ming Zhou, Jian Ma, Xi Huang, Xu Wu, Yong Li, Gui-Yuan Li, Zhao-yang Zeng, Wei Xiong

**Affiliations:** 1 The Key Laboratory of Carcinogenesis of the Chinese Ministry of Health, Hunan Cancer Hospital and The Affiliated Cancer Hospital of Xiangya School of Medicine, Central South University, Changsha, 410013 Hunan China; 2 The Key Laboratory of Carcinogenesis and Cancer Invasion of the Chinese Ministry of Education, Cancer Research Institute and School of Basic Medical Science, Xiangya School of Medicine, Central South University, Changsha, 410078 China; 3 Department of Molecular Genetics, University of Toronto, Toronto, ON M5S 1A8 Canada; 4 Department of Chemistry, University of North Dakota, Grand Forks, North Dakota 58202 USA; 5 Department of Cancer Biology, Lerner Research Institute, Cleveland Clinic, Cleveland, OH 44195 USA

**Keywords:** Cancer metabolism microenvironment, Regulatory T cells, Hypoxia, Low pH, Signaling pathway

## Abstract

Recent studies have shown that on one hand, tumors need to obtain a sufficient energy supply, and on the other hand they must evade the body’s immune surveillance. Because of their metabolic reprogramming characteristics, tumors can modify the physicochemical properties of the microenvironment, which in turn affects the biological characteristics of the cells infiltrating them. Regulatory T cells (Tregs) are a subset of T cells that regulate immune responses in the body. They exist in large quantities in the tumor microenvironment and exert immunosuppressive effects. The main effect of tumor microenvironment on Tregs is to promote their differentiation, proliferation, secretion of immunosuppressive factors, and chemotactic recruitment to play a role in immunosuppression in tumor tissues. This review focuses on cell metabolism reprogramming and the most significant features of the tumor microenvironment relative to the functional effects on Tregs, highlighting our understanding of the mechanisms of tumor immune evasion and providing new directions for tumor immunotherapy.

## Background

A tumor is a type of polygenic disease in which malignant cell proliferation is caused by a variety of oncogenic factors that promote abnormal gene expression and regulation in the body [1]. It differs from the body’s normal tissue cells, and is mainly characterized by the following ten characteristics: unlimited proliferation, resistance to apoptosis, evasion of growth inhibitory factors, invasion and metastasis, stimulation of angiogenesis, continuous production of proliferative signals, resistance to cellular energy metabolism, genomic instability, evasion of immune suppression, and enhancement of tumor-related inflammatory responses [2–4]. Acquisition of these characteristics is inseparable from changes in the cells themselves and the influence of the surrounding environment. The internal environment in which tumor cells grow is called the tumor microenvironment, which consists of the extracellular matrix, myofibroblasts, cytokines, fibroblasts, neuroendocrine cells, adipocytes, immune-related cells, and vasculature [5–8].Tumor cells can affect the physicochemical properties, components, and cytokines of the tumor microenvironment, making it more conducive to their growth, metastasis, and invasion [9–13]. At the same time, changes in tumor microenvironment play an important role in all stages of tumor development, inhibiting or promoting tumor growth. The interaction between the two affects the whole process of tumor growth, and mutual adaptation maintains the dynamic balance of tumor microenvironment [14–19].

Owing to changes in their physiological characteristics, tumor cells promote a series of changes in the tumor microenvironment [20–23]. Rapidly growing tumor cells and mesenchymal cells lead to a decrease in local oxygen content and thus to a microenvironment in an oxygen-starved state, with the most severe hypoxia in the center of the tumor and a gradient decrease from external to internal oxygen content. Tumor growth to 1–2 mm in diameter stimulates local angiogenesis [24], and local hypoxia induces the expression of vascular endothelial growth factor (VEGF), which in turn promotes the formation of blood vessels in tumors [24, 25]. However, owing to the abnormal tumor microenvironment, new blood vessels often have uneven distribution, large capillary distance, short arteriovenous circuit, incomplete endothelial cells, and ruptured basal membranes [26]. Therefore, these localized microvessels cannot improve the state of hypoxia. With changes in the tumor microenvironment, the metabolism of tumor cells is also quite different from that of normal cells. The most significant difference is the Warburg effect, in which, compared with normal cells, tumor cells obtain energy through anaerobic glycolysis even under oxygen-rich conditions [27, 28]. At the same time, hypoxia causes high expression levels of hypoxia-inducible factor (HIF) in cells. This in turn promotes the expression of glycolysis-related proteins [29], and changes in glucose metabolism lead to the massive production of lactic acid metabolites. An oncogene such as *c-myc* is activated by tumor cells and up-regulates the expression of lactate dehydrogenase A (LDH-A) and promotes the conversion of pyruvate to lactic acid [30]. This leads to an increase in lactate content and low pH in tumor cells. To avoid the effects of intracellular lactic acid on basal metabolic activity, the efficiency of the monocarboxylate transporter (MCT) on the cell membrane is increased, removing excess lactic acid from the cell [31]. This causes the tumor microenvironment to be at a low pH. HIF can induce high expression levels of carbonic anhydrase (CA) in tumor cells, which catalyzes the reaction of CO_2_ with H_2_O to generate carbonic acid [32]. Stimulation of various types of carcinogens and cytokines leads to disturbances in the acid-base balance in tumor cells. Tumor cells promote an efflux of intracellular hydrogen ions by up-regulating hydrogen ion-related transport proteins in the cell membrane [33]. The above changes will further exacerbate the low pH of the microenvironment. In addition, de novo synthesis of fatty acids in tumor cells is also enhanced [34, 35]. Glutamine metabolism is enhanced, and the metabolism of other amino acids and nucleic acids also changes [36, 37]. These metabolic changes promote tumor cell proliferation, metastasis, and invasion [38–40] and also cause changes to the microenvironment of the tumor, affecting other cells infiltrating the tumor tissue. Metabolic changes occur not only in tumor cells, but also in immune cells infiltrated in the tumor tissue that undergo metabolic reprogramming to accommodate functional changes.

During proliferation and metastasis, tumor cells need to obtain enough energy and reaction substrates to satisfy their own metabolism [41–44], and it is necessary to escape the surveillance and elimination of abnormal tissue cells by the immune system [45]. The immune evasion of tumor cells occurs in two ways: covering the self-antigens to hide or remove the target that can be recognized by the host immune system, also known as antigen coverage; or secretion of immunosuppressive cytokines to inhibit the immune effector cells or induction of suppressive immune cells to exert immunosuppressive effects [46]. Tregs belong to a T cell subgroup with regulatory immune functions. These cells participate in the immune escape of tumor cells mainly through the induction of immune incompetence and immunosuppression [47]. Clinical studies have found a large infiltration of Tregs in tumor tissues of patients, and their amount is closely related to the prognosis of patients [48, 49]. Some tumors can be treated by reversing local Tregs levels in tumor tissue [50, 51]. Animal experiments showed that tumor growth is positively correlated with Tregs local content in tumors, and removal of Tregs can effectively enhance the body’s resistance to tumors [52, 53]. Tregs have a strong ability to infiltrate and accumulate in tumor tissues. The chemokines in the tumor microenvironment can bind to the corresponding receptors on the surface of Tregs, recruit Tregs to the tumor tissues, and exert immunosuppressive effects [54]. Antigens in tumor tissues can promote the production of Tregs after dendritic cell processing and extraction [55]. Tumor cells secrete cytokines to directly induce the transformation of T cells into Tregs [56], or indirectly promote the production of Tregs by inducing the maturation of myeloid dendritic cells that secrete immunosuppressive factors [57].

Tregs are abundantly present in tumor tissues and mainly mediate immunosuppressive effects. An increasing number of studies has focused on the metabolic reprogramming of Tregs in tumor tissues and whether tumors and Tregs can influence each other, and on how to modulate Tregs or the biologically active substances or cells that affect these T cells in the tumor microenvironment to improve the patient’s prognosis. Therefore, this review focuses on the metabolic reprogramming of Tregs in tumor tissues and the effects of the physicochemical properties of the tumor microenvironment on the generation, proliferation, and infiltration of Tregs and its mechanisms of action. Advances in understanding of the complex regulatory networks of tumor immune evasion provide a theoretical basis and new biological targets for clinical treatment.

## The physiological function of Tregs

**Fig. 1:**
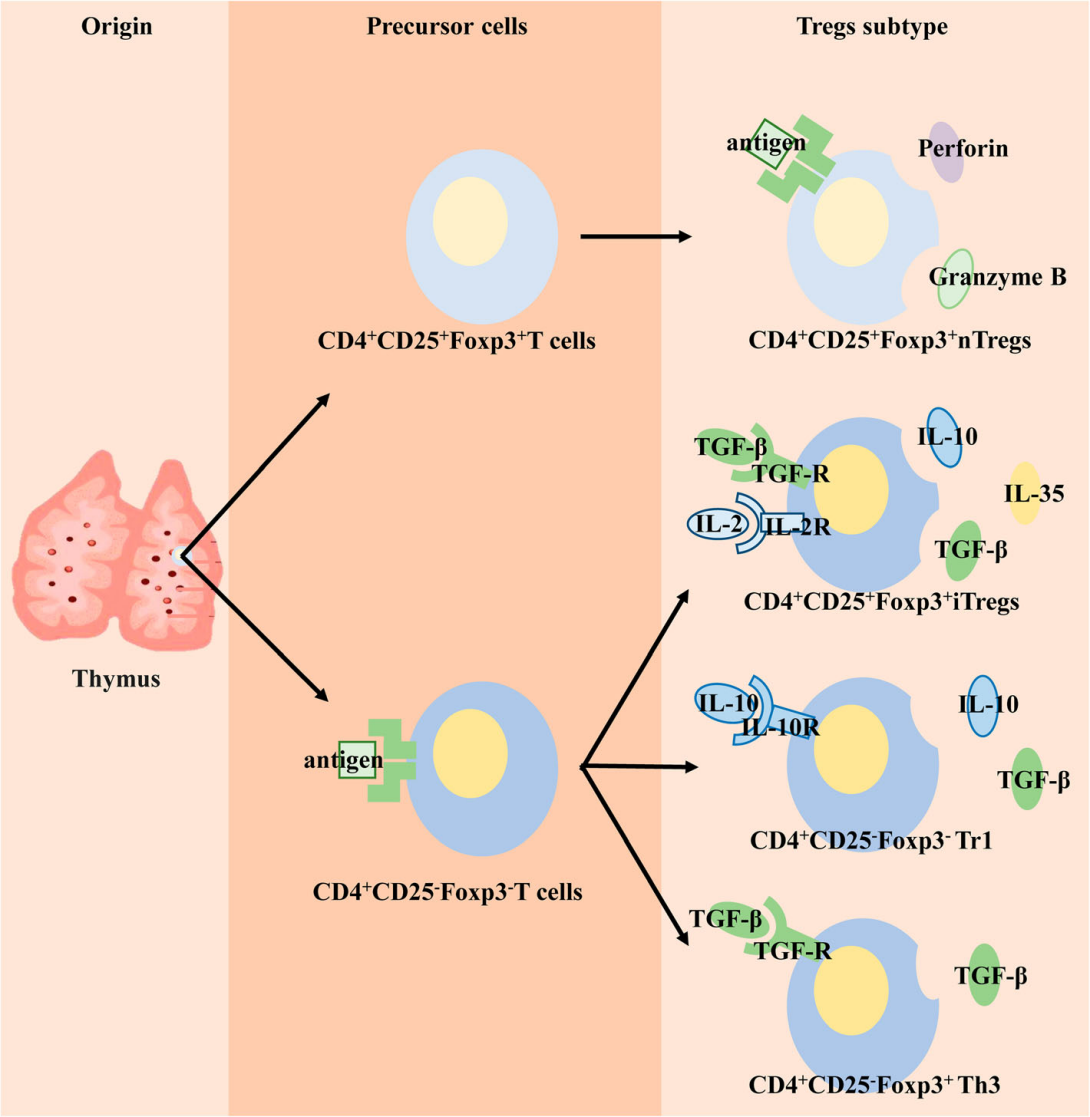
Differentiation of Tregs. nTregs: mature after thymus undergoes selection and exerts an immune function in the periphery. iTregs: peripheral T cells stimulated by antigen are converted to different Tregs subtypes by different inhibitory cytokines, secreting different cytokines to play immunosuppressive roles

Tregs are a subset of T cells that express the interleukin (IL)-2 receptor CD25 and the forkhead/flanking helix nuclear transcription factor (Foxp3), and were first discovered in 1995 [58]. Tregs in the body are divided into two groups: natural Tregs (nTregs), which mature after positive and negative selection of the thymus and exert immunosuppressive effects in peripheral blood and lymphoid tissues; and inducible Tregs (iTregs), which originate after T cells receive antigen stimulation and are transformed by inhibitory cytokines [59], having differentiated into different subtypes by different cytokines and playing different biological roles [60] (Fig. 1/Table. 1).

**Table 1:** Treg subtypes and functions

Treg classification	Treg subtype	Stimulus factor	Secretion factor	Biological role
Natural Tregs (nTregs)	CD4 + CD25 + Foxp3 + nTregs	Antigen	Perforin, Granzyme B	Curbs the occurrence of autoimmune diseases and induces transplant tolerance
Inducible Tregs (iTregs)	CD4 + CD25 + Foxp3 + iTregs	TGF-β, IL-2	IL-10, IL-35, TGF-β	Maintains the stability of the internal environment, curbs the occurrence of autoimmune diseases, anti-tumor immunity, and anti-infective immunity
	CD4 + CD25-Foxp3- Tr1	IL-10	IL-10, TGF-β	Inflammatory autoimmunity
	CD4 + CD25-Foxp3+ Th3	TGF-β	TGF-β	Participates in oral tolerance and mucosal immunity

Foxp3 is a key transcription factor of Tregs, mainly related to the development and function of these cells. Foxp3 regulates the immunosuppressive function of Tregs mainly through transcriptional co-regulatory proteins, including transcription factors, co-repressors, co-activators, histones, and chromatin remodeling factors, which combine to form protein complexes to dynamically regulate the expression of related genes [61–65].

**Fig. 2:**
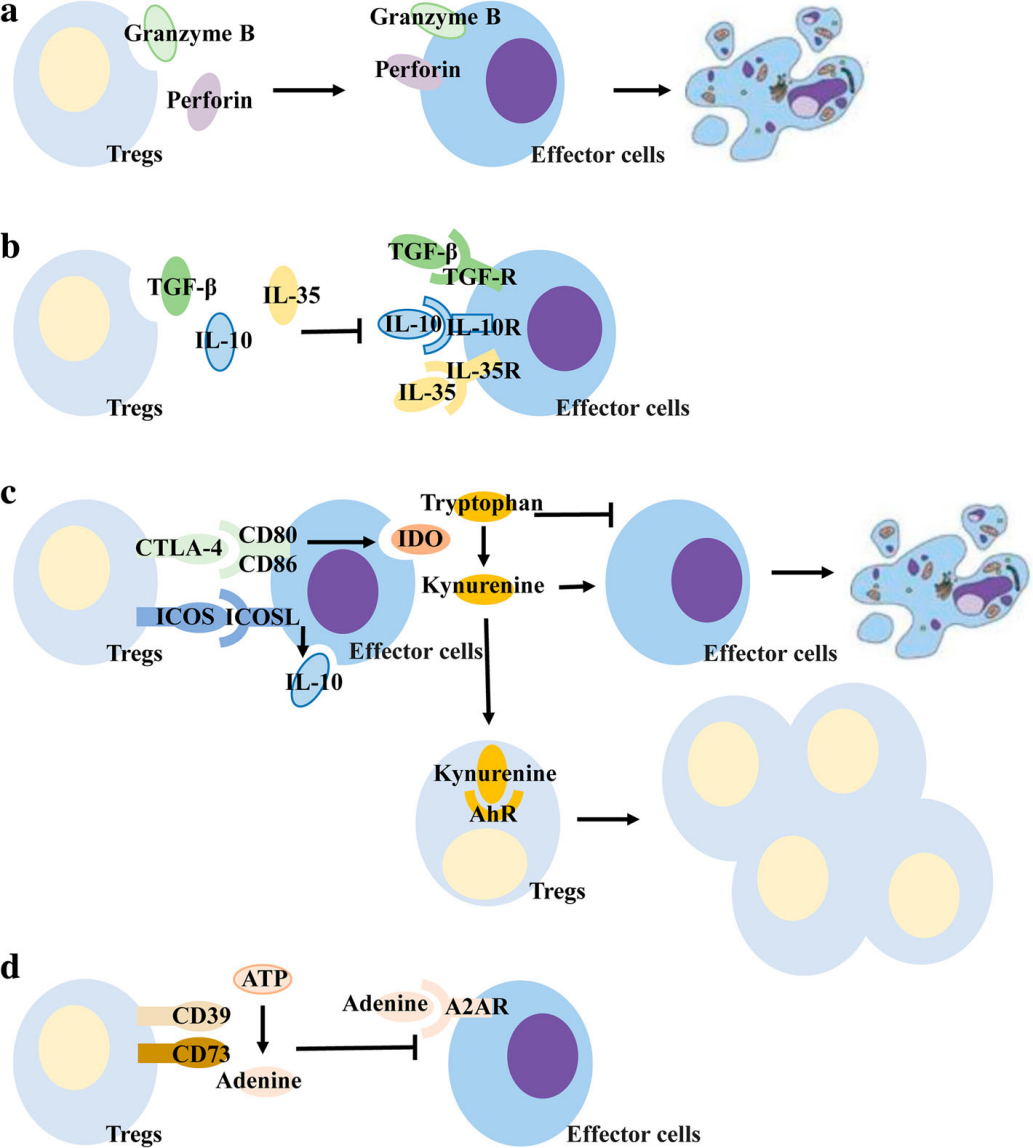
Immunosuppression of Tregs. (**a**)Tregs secrete granzyme B and perforin that act on effector cells and cause apoptosis. (**b**)Tregs secrete inhibitory cytokines that bind to receptors on the surface of effector cells and inhibit the immune response. (**c**) CTLA-4 on the surface of Tregs competes with CD80/CD86 on the surface of effector cells to inhibit their immune function and promote the secretion of IDO; IDO degrades tryptophan in tissues to kynurenine; deletion of tryptophan leads to effector cell apoptosis; and kynurenine promotes effector cell apoptosis and acts on Tres’s AhR to promote its proliferation. ICOS on the surface of Tregs binds to ICOSL on the surface of effector cells, promoting the effector cell secretion inhibitory cytokine IL-10. (**d**) CD73/CD39 on the surface of Tregs convert ATP in tissues to adenine, which binds to receptors on the surface of effector cells and inhibit their immune function

Tregs exert immunosuppressive effects by inhibiting effector T cells and dendritic cells mainly through the following pathways: (a) secretion of perforin and granzyme B that act directly on effector cells promoting apoptosis [66]; (b) secretion of inhibitory cytokines such as transforming growth factor (TGF)-β, IL-10, and IL-35 that bind to immune cells and result in immunosuppressive effects [67]; (c) by interacting with target cells via its surface receptors, such as cytotoxic T lymphocyte-associated antigen (CTLA)-4, to inhibit immune function by binding to CD80/CD86 on the surface of effector cells [68], thereby inducing effector cells to secrete indoleamine 2,3 dioxygenase (IDO), which catalyzes the conversion of tryptophan in tissues to kynurenine. The loss of tryptophan inhibits the activation of effector cells and induces apoptosis [69] . The increased kynurenine not only inhibits the immune function of effector cells, but also regulates Treg proliferation and differentiation by acting on the aromatic hydrocarbon receptor (AhR) [70]. Specifically, invasive Tregs in pancreatic cancer can bind to CD80/CD86 on the surface of dendritic cells via surface CTLA-4 to inhibit the function of dendritic cells in tissues, which, in turn, affects the function of cytotoxic CD8+ T cells to suppress immune responses in pancreatic cancer [71]. Tregs surface also expresses costimulatory molecules belonging to the CD28 family----induced costimulatory molecules (ICOS) [72]. ICOS binds to ICOSL, a receptor on the surface of effector cells, and promotes the secretion of inhibitory cytokines, especially IL-10 [73]; and (d) conversion of intercellular ATP to adenine through the cell surface receptors CD73 and CD39, with adenine binding to the adenosine receptor A2AR on the surface of effector cells and inhibiting its proliferation and immune function [74]. (Fig. 2).

## Effects of metabolic reprogramming on Tregs

As the tumor microenvironment undergoes a series of changes in physical and chemical properties, Tregs infiltrating tumor tissues are also metabolically reprogrammed to meet the needs of differentiation, proliferation, and function. Metabolic reprogramming is primarily involved in glycolysis, fatty acid oxidation (FAO), and oxidative phosphorylation (OXPHOS). Glycolysis is a process in which glucose is degraded in the cytoplasm to produce pyruvic acid and then reduced to lactic acid in an oxygen-deficient state. It can quickly provide the required energy to the cells, and the metabolites can participate in the growth and function of the cells as substrates for other reactions. FAO is a process in which intracellular fatty acids are oxidatively decomposed into CO_2_ and H_2_O by the action of related enzymes and release a large amount of energy. OXPHOS mainly occurs in mitochondria, the process by which the metabolically produced active substance is electronically coupled through the respiratory chain to ultimately couple to form ATP. These metabolic changes interact to affect the immune function of Tregs.

### Reprogramming of glucose metabolism affects Treg chemotaxis and immune function

In activated Tregs, the surface molecule CD28 is stimulated to activate the downstream PI3K protein, which stimulates downstream mTORC2 activation and ultimately promotes glucokinase (GCK) expression. On the one hand, GCK can bind to actin to promote the rearrangement of the cytoskeleton and promote the migration of Tregs into tumor tissues. On the other hand, as an isozyme of hexokinase, it can promote glycolysis in Tregs and provide energy for the movement of the cytoskeleton [75].

**Fig. 3:**
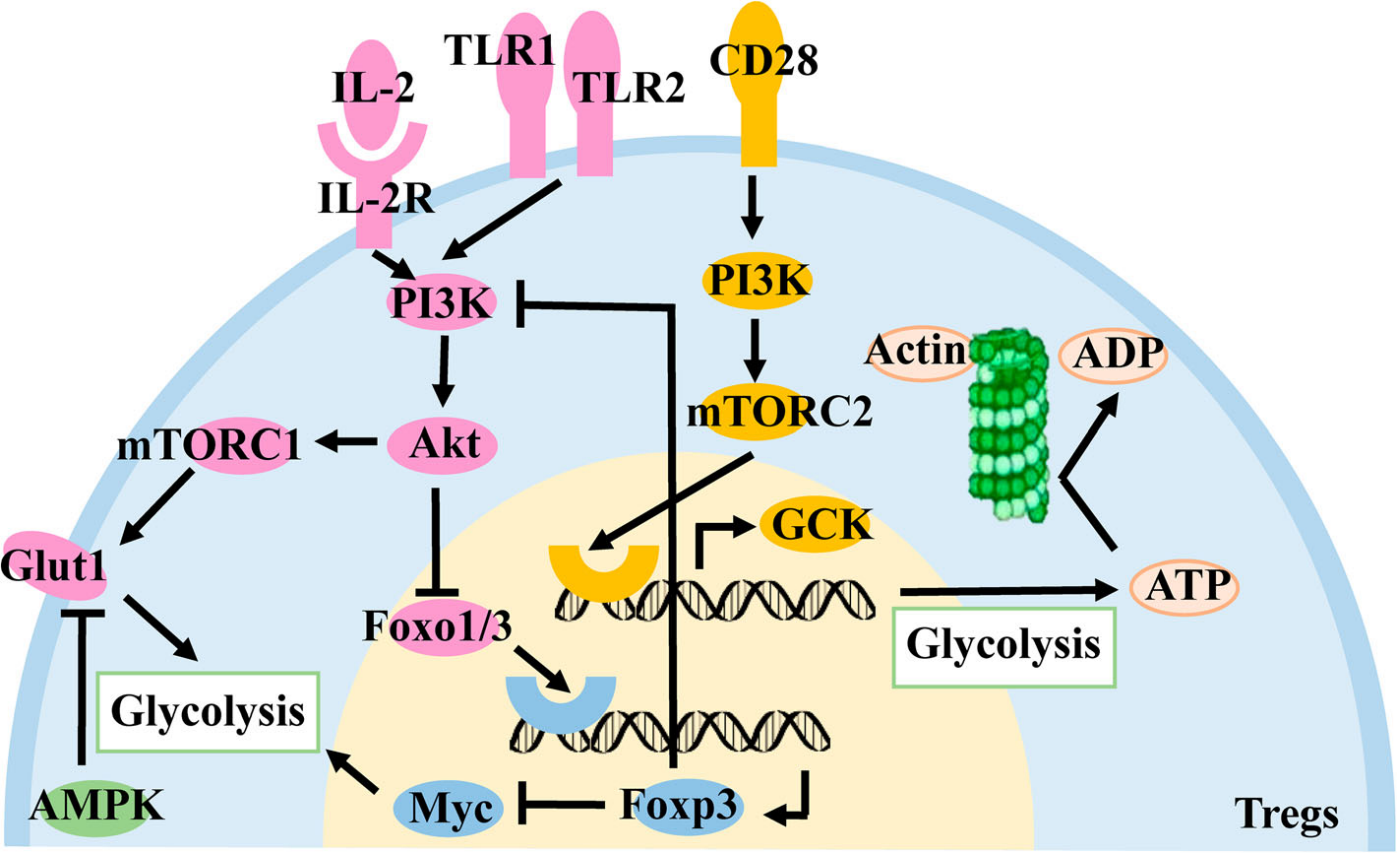
Effect of glycolysis on Tregs. CD28 on the surface of Tregs is activated to promote GCK expression via PI3K/mTORC2, GCK, and actin, which can promote cytoskeletal remodeling, and GCK can promote glycolysis to provide energy. Upon stimulation of TLR1/2 or IL-2R on the Treg surface, activation of the PI3K/AKT/mTORC1 signaling pathway promotes Glut1 expression on the cell membrane surface and promotes glycolysis to promote proliferation. Akt inhibits the function of Tregs by inhibiting the expression of Foxp3, which in turn inhibits the transcription factor Foxo1/3. Foxp3 inhibits glycolysis by inhibiting the signaling pathway of PI3K/AKT/mTORC1 and the expression of Myc. AMPK in Tregs inhibits glycolysis by suppressing mTORC1

The Toll-like receptor (TLR) on the surface of Tregs is also involved in metabolic reprogramming. After stimulation by the microenvironment, TLR1 and TLR2 activate the downstream PI3K-Akt-mTORC1 signaling pathway, resulting in the expression of glucose transporter 1 on the cell membrane. Glut1 can transport extracellular glucose into cells for glycolysis, providing the required energy for Tregs, which is beneficial to their proliferation, but the activation of this signaling pathway impairs the immunosuppressive function of these cells [76]. IL-2 and its receptors are also involved in the activation of the PI3K-Akt-mTORC1 signaling pathway [77]. The inhibitory effect on Tregs is a result of the expression of the transcription factor Foxo1/3 required for inhibition of Foxp3 expression by Akt in the signaling pathway [78, 79]. However, FOXp3 can inhibit Tregs glycolysis by inhibiting the PI3K-Akt-mTORC1 signaling pathway through feedback regulation [76], and, at the same time, inhibits the expression of the transcription factor Myc [80]. As a growth factor, Myc can induce cells to undergo metabolic reprogramming such as the shift from glycolysis [81], thus indirectly inhibiting glycolysis in the cell. AMP kinase (AMPK) inhibits the expression of Glut1 and glycolysis in Tregs by inhibiting the mTORC1 signaling pathway [82]. It is speculated that glycolysis may play different roles in different functional phases of Tregs. (Fig. 3).

### Lipid metabolism reprogramming promotes Treg immune function

AMPK expressed in Tregs is not only involved in the reprogramming of glucose metabolism, but also promotes FAO by increasing the expression of FAO’s key enzyme, carnitine palmitoyltransferase-1A (CPT1A) [83]. PD-1 on the surface of Tregs can also promote FAO by increasing the expression of CPT1A [84]. Treg’ FAO process promotes Treg proliferation, stability, and immunosuppressive function.

### Oxidative phosphorylation promotes Tregs immune function

**Fig. 4:**
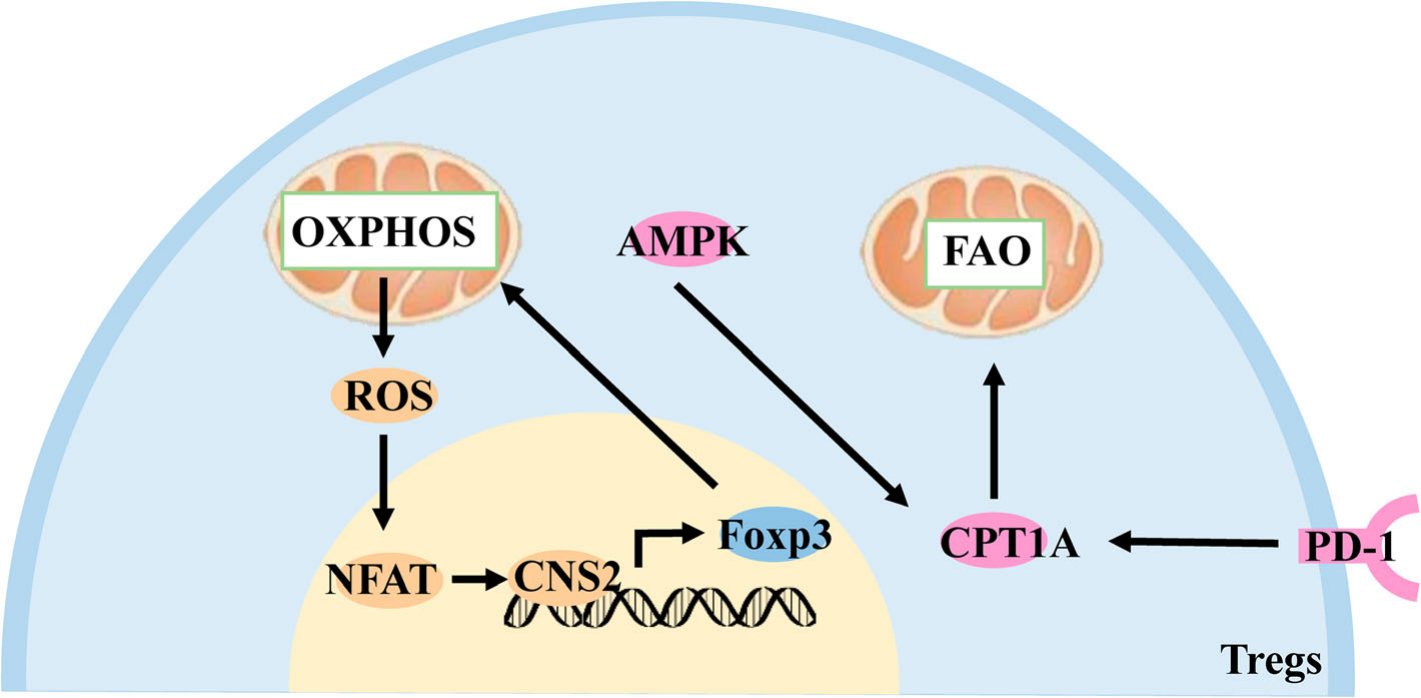
Effect of FAO and OXPHOS on Tregs. AMPK in Tregs promotes FAO by increasing the expression of CPT1A. PD-1 on the surface of Tregs also promotes FAO by increasing CPT1A expression. Foxp3 promotes OXPHOS by increasing the expression of mitochondria-associated proteins, ROS, a by-product of OXPHOS, stabilizes NFAT in the nucleus and binds to CNS2 to promote Foxp3 expression

Foxp3, the most important transcription factor in Tregs, promotes mitochondrial OXPHOS by increasing the expression of mitochondria-associated genes and mitochondrial electron transport system proteins. These changes not only provide the energy needed for Tregs to exert immunosuppressive function, but also consume long-chain fatty acids in cells and protect from fatty acid-induced apoptosis [85]. Metabolic reactive oxygen species (ROS) produced during the OXPHOS process also stabilize the transcription factor-activated T cell nuclear factor (NFAT) in the nucleus [86], which binds to the non-coding sequence 2 (CNS2) of the enhancer upstream of the Foxp3 gene [87] and promotes its expression. (Fig. 4).

Treg’ metabolic reprogramming mainly inhibits glycolysis and promotes FAO and OXPHOS, so that cells can adapt to changes in a tumor microenvironment with low glucose and high lactic acid and promote cell proliferation, differentiation and immune function.

## Effects of microenvironmental changes on Tregs

Hypoxia in the microenvironment leads to high expression levels of the transcriptional regulatory factor, HIF, in tissue cells, which are positively correlated with the degree of hypoxia [88]. HIF is a transcriptionally active heterodimer consisting of an α-subunit and a β- subunit. HIF-α has three subtypes: HIF-1α, HIF-2α, and HIF-3α [89]. HIF binds to hypoxia response elements (HREs) to promote the transcription of related downstream genes and to express the encoded protein to respond to the effects of hypoxia on cells [90].

The acidic microenvironment not only promotes the release of active substances such as immunosuppressive cytokines by affecting the intracellular metabolic environment, but also affects the biological and physical properties of tumor cell membranes to promote exosome release [91]. Exosomes are small vesicles containing complex RNA and proteins for information exchange between cells [92].Caveolin-1 can also promote the release of tumor-derived exosomes under acidic conditions [91]. Similarly, microenvironment hypoxia may promote the secretion of exosomes by tumor cells by activating the HIF signaling pathway [93].

### Microenvironment chemotactic signals recruit Tregs to tumor tissue

Changes in the tumor microenvironment can affect the levels of infiltrating cell-associated chemokines in tumor cells and tissues. These chemokines, in turn, recruit Tregs to tumor tissues by chemotaxis to exert immunosuppressive effects.

Chemokines are a class of cytokines that are expressed on the cell surface or secreted into the cell mesenchyme and have a chemotactic effect on certain cell types. Chemotaxis is exerted by binding to the corresponding chemokine receptors on the cell surface. Chemokine receptors are G protein-coupled receptors that are widely expressed in various cells and are also divided into four families based on the corresponding ligands. A cross-activation between receptor and ligand is also observed.

Under hypoxia, ovarian cancer cell lines express a large number of CCL28 receptors and recruit Tregs to tumor tissues. HIF-1α inhibitors reduce CCL28 levels and decrease its chemotactic ability to recruit Tregs. It is speculated that ovarian cancer cells promote the expression of CCL28 on the cell surface through the HIF signaling pathway during hypoxia, and CCL28 interacts with CCR10 on the surface of Tregs to recruit it by chemotaxis into the tumor tissue and exert immunosuppressive effects [94]. The same findings were observed in liver cancer cell lines [95].

Under hypoxic conditions, basal-like breast cancer cells exert immunosuppressive effects by affecting the expression of chemokine receptors on the surface of Tregs. HIF acts on HREs to promote the expression of CXCR4 on the surface of Tregs cells, which in turn bind to CXCL12 on the surface of tumor cells, and then Tregs are recruited by chemotaxis into tumor tissues. In addition to its direct action, HIF-1α can also promote the expression of downstream Foxp3 by binding to HREs, and indirectly promote the expression of CXCR4 by acting on regulatory sequences upstream of the CXCR4 transcription initiation site [96]. This phenomenon was also observed in ovarian cancer [97], lung adenocarcinoma [98], and malignant mesothelioma [99].

Nasopharyngeal carcinoma cells secrete exosomes under the influence of the microenvironment to recruit Tregs by chemotaxis. Tumor-derived exosomes contain CCL20, which is released in the interstitial space and then binds to CCR6 on the surface of Tregs. These cells are recruited by chemotaxis to the tumor tissue and exert immunosuppressive effects [100].

**Fig. 5:**
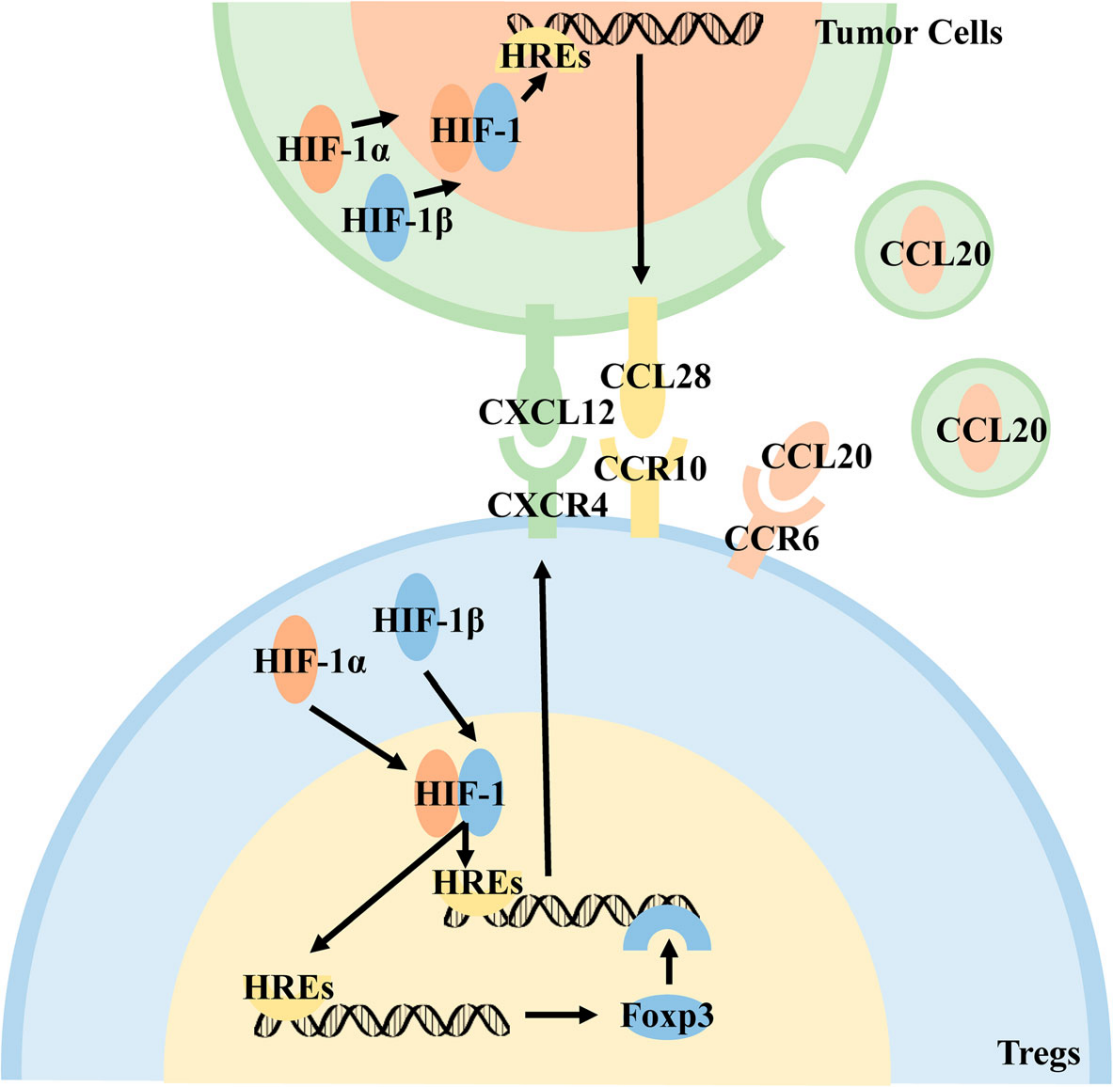
The microenvironment recruits Tregs to tumor tissue by chemotaxis. Hypoxia promotes the expression of chemokines on tumor cells and Tregs surface through the HIF signaling pathway, and recruits Tregs by chemotaxis to the tumor tissue. Low pH microenvironment promotes the secretion of chemokine-containing exosomes from tumor cells that recruit Tregs to tumor tissue by chemotaxis

The tumor microenvironment can directly recruit activated Tregs by chemotaxis into tumor tissues, or recruit T cells into tumor tissues that differentiate into Tregs under the stimulation of certain cytokines to exert immunosuppressive effects (Fig. 5).

### Microenvironment promotes differentiation of T cells into Tregs

A large number of T cells are found infiltrating in the tumor tissue. These T cells can differentiate into immune cells of several subtypes under the influence of different cytokines in the microenvironment, thus exerting different immune effects.

HIF-1α in CD4^+^ T cells is stably expressed under hypoxia and binds to HIF-1β in the nucleus. Subsequent binding to conserved HIF-1 binding sites on HREs promotes downstream expression of Foxp3 [101, 102]. As the main transcription factor of Tregs, Foxp3 can regulate most gene expression related to Treg differentiation, proliferation, and immune function.

The above process is further improved [103]. The downstream regulatory genes of HIF-1 also include TGF-β, and hypoxia can promote the expression of TGF-β in CD4+ T cells. On the one hand, TGF-β is secreted into the cell mesenchyme and binds to the corresponding receptors on the cell membrane to activate downstream signaling pathways and regulate cell differentiation. On the other hand, TGF-β can inhibit the key enzyme PHD2 [104] in the proteasome-mediated degradation of HIF-1α, and thus indirectly promote the expression of HIF-1α in T cells.

Hypoxia not only directly promotes the conversion of T cells into Tregs, but also indirectly promotes the differentiation of T cells into Tregs by affecting the secretion of cytokines or enhancing the expression of cell surface molecules in cells infiltrating tumor tissues. Immunohistochemistry showed co-localization of HIF-1α and TGF-β in gastric cancer, suggesting that HIF-1 combined with HREs also promote the expression of downstream TGF-β molecules in gastric cancer cells. Subsequently, TGF-β is secreted into the cytoplasm and binds to the TGF-β receptor on the T cell membrane, promoting the downstream phosphorylation of SMAD3. The phosphorylated SMAD3 binds to SMAD4 and then the complex enters the nucleus, recruiting the coactivator CBP/p3000 and then binding to Foxp3 regulatory sequences to promote Foxp3 expression [105].

**Fig. 6:**
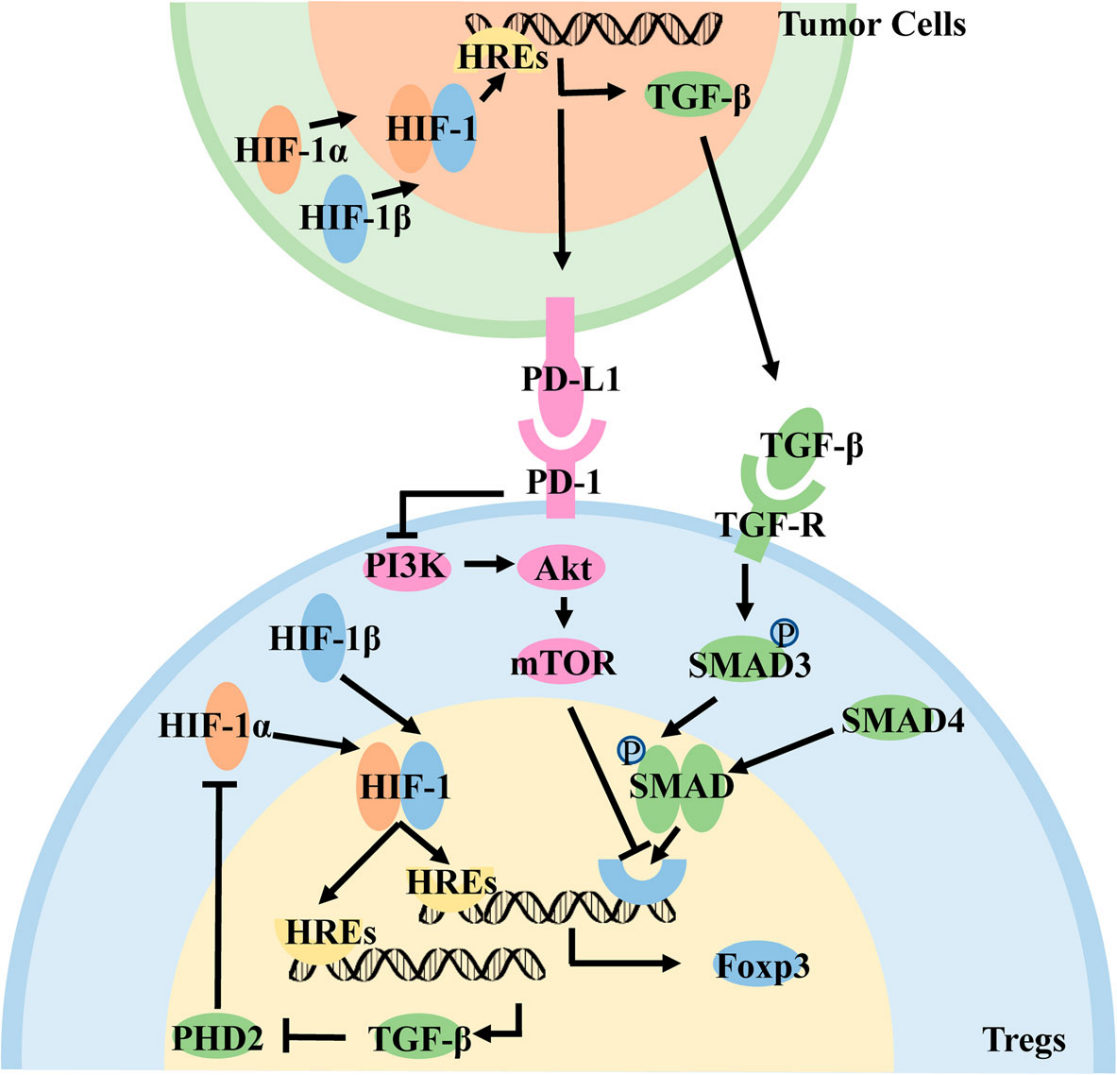
Hypoxia induces differentiation of T-cells to Tregs. T cells directly promote the expression of Foxp3 through the HIF signaling pathway. T cells promote the expression of TGF-β through HIF signaling, inhibit the key enzyme PHD2 for HIF-1α degradation, and indirectly promote the expression of Foxp3. Tumor cells promote the expression of TGF-β through the HIF signaling pathway, act on the receptors on the surface of T cells, activate the downstream SMAD signaling pathway, and promote the expression of Foxp3.Tumor cells promote the expression of PD-L1 on the cell surface through the HIF signaling pathway, bind PD-1 on the surface of T cells, inhibit the downstream AKT and mTOR pathways, and promote the expression of Foxp3

Programmed cell death protein-1(PD-1) is an active molecule that is widely expressed on the surface of T cells and B cells. It can bind to programmed death-ligand-1(PD-L1) and initiate downstream signaling pathways, regulating the activation of immune cells. Under hypoxic conditions, HIF-1α in tumor cells, bone marrow-derived suppressor cells, and dendritic cells promotes the expression of downstream PD-L1 by acting on HREs. PD-L1 binds to PD-1 on the surface of T cells, causing the dephosphorylation of the downstream protein PI3K, which in turn inhibits the activation of downstream AKT/mTOR to promote the expression of Foxp3 [106]. In renal cell carcinoma, hypoxia mainly promotes the expression of PD-L1 by stabilizing the expression of HIF-2α, and then PD-1 binds to the surface of T cells to promote differentiation into Tregs [107] (Fig. 6).

Tumor-derived exosomes contain TGF-β and IL-10 [108–110] and release their contents after secretion from cells. TGF-β binds to receptors on the surface of T cells and differentiates into Tregs by activating the downstream SMAD signaling pathway. However, binding of IL-10 to the IL-10R on the surface of T cells activates downstream JAKs and activates the transcription factor STAT3 through phosphorylation of related molecules. Acting on STAT binding elements promotes the expression of Foxp3, which differentiates T cells into Tregs. Tumor-derived exogenous TGF-β may have a greater impact on Tregs because IL-10 can only induce T-cell differentiation into Tregs, whereas TGF-β can promote Tregs proliferation in addition to inducing differentiation.

**Fig. 7:**
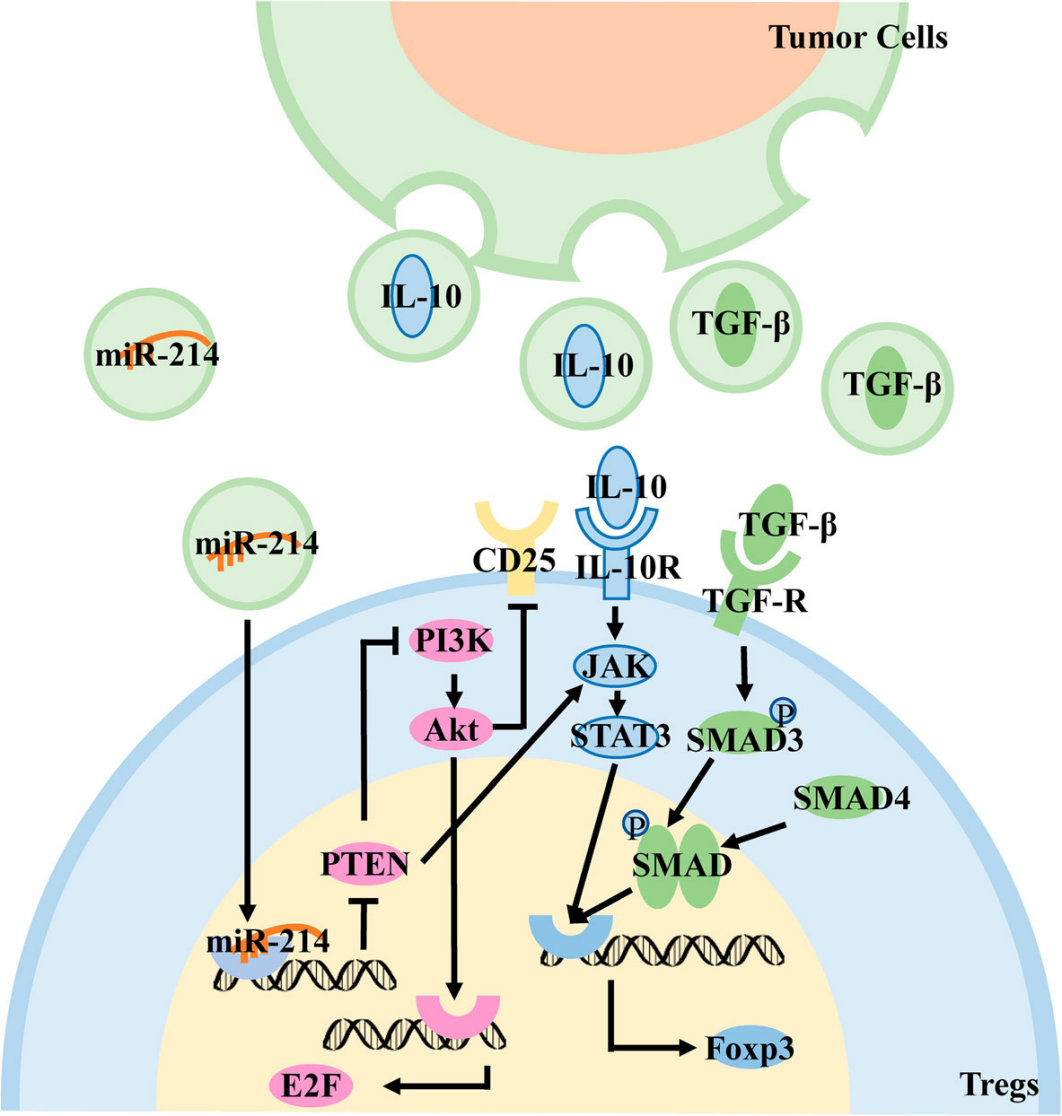
Low pH induces differentiation of T cell into Tregs. Tumor-derived exosomes contain TGF-β, which acts on the receptor on the surface of T cells to activate the SMAD signaling pathway and promote the expression of Foxp3. Tumor-derived exosomes contain IL-10, which acts on receptors on the T cell surface to activate the JAK/STAT signaling pathways and promote Foxp3 expression. Tumor-derived exosomes contain miR-214, which enters Tregs by endocytosis, inhibits the expression of PTEN protein, and then promotes the expression of the cycle-related transcription factor E2F by activating the PI3K signaling pathway and promote Tregs proliferation. PTEN maintains the stability of Tregs by inhibiting the activation of PI3K/Akt to stabilize the expression of CD25 and promotes the induction of the JAK-STAT signaling pathway to maintains the stability of Tregs

In addition to immunosuppressive cytokines, tumor-derived exosomes also contain microRNAs (miRs) that regulate cell differentiation. Exosomes containing miR-214 can directly bind to the T cell membrane, transferring miR-214 into T cells by endocytosis, which not only promotes the secretion of IL-10 by T cells, but also binds to the 3’UTR region of PTEN, reduces the expression of PTEN protein in cells, and activates the PI3K-Akt signaling pathway [111]. This signaling pathway enables the intracellular accumulation of the cycle-associated transcription factor E2F to promote the proliferation of Tregs [112]. PTEN is also a major regulator of Treg stability. A large number of PTEN deletions in Tregs affect PI3K-Akt regulation, leading to CD25 deletion, and the accumulation of Foxp3 + CD25-T cells ultimately leads to the loss of Foxp3 expression in these cell populations; therefore, PTEN regulates Treg lineage homeostasis and stability by inhibiting the PI3K-Akt signaling pathway [113]. Moreover PTEN can also promote the induction of the JAK-STAT signaling pathway to promote the expression of FOXp3 [114] (Fig. 7).

### The metabolite, RA, promotes differentiation of T cells into Tregs

**Fig. 8:**
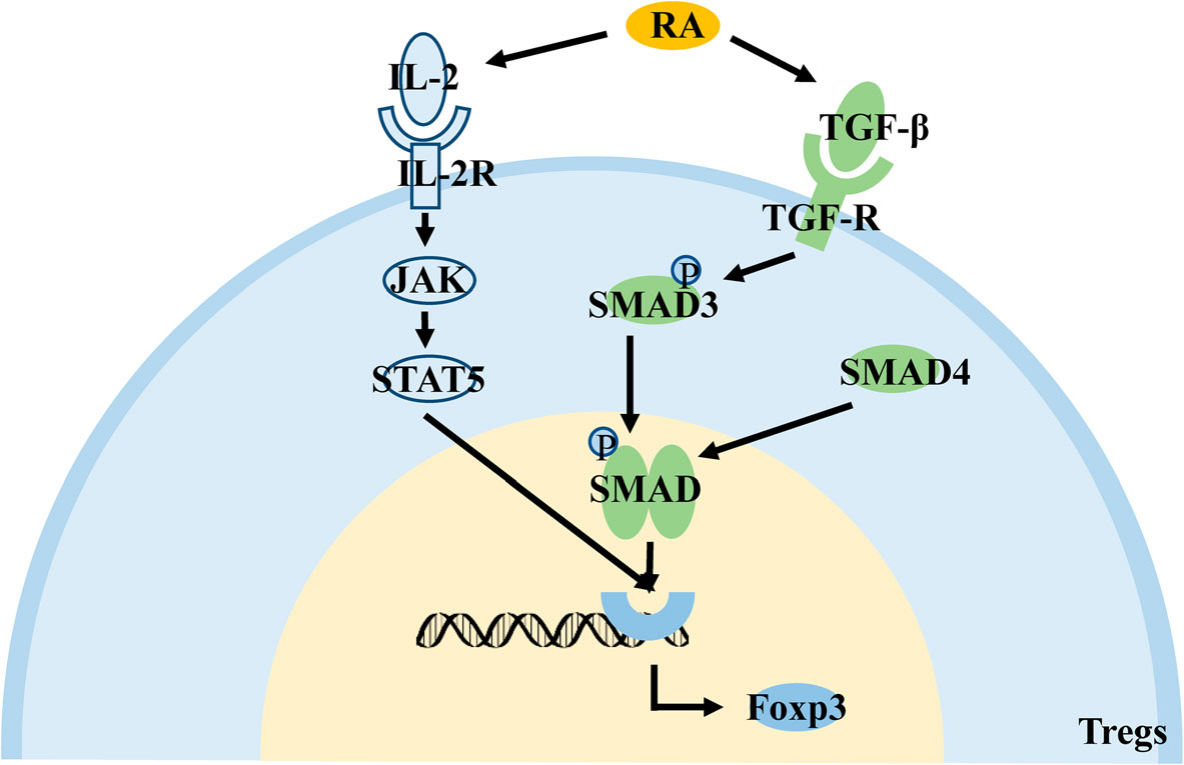
Effects of the metabolite, RA, on Tregs. RA in the microenvironment promotes the expression of Foxp3 by promoting the activation of the downstream JAK/STAT5 signaling pathway by IL-2 and the activation of the downstream SMAD signaling pathway by TGF-β, indirectly promoting the differentiation and proliferation of Tregs

Metabolites in the microenvironment can also affect the function of Tregs. Retinoic acid (RA) is a metabolite of vitamin A in dendritic cells [115]. The ability of RA to directly induce the differentiation of T cells into Tregs is weak; however, it can significantly enhance the efficiency of TGF-β and IL-2 to promote the differentiation of T cells into Tregs [116]. As an important molecule that promotes the function of Tregs, IL-2 can promote the expression of Foxp3 by activating the downstream JAK/STAT5 signaling pathway by binding to the IL-2 receptor [117]. RA indirectly affects the quantity and function of infiltrating Tregs in tumor tissues. (Fig. 8).

## Conclusions

Owing to their rapid proliferation and invasion and metastasis characteristics, tumor cells have large differences in their metabolic activities from normal cells. There are many cell types that infiltrate the microenvironment in which the tumor cells are located, including immune cells and mesenchymal cells [118]. Changes in metabolic activity and an increase in infiltrating cells lead to a change in the physicochemical properties of the tumor microenvironment, the most notable of which are hypoxia and pH reduction. Metabolic reprogramming and microenvironmental changes will cause changes in cell-associated transcription factors and secretory substances, which will affect the occurrence and development of tumors.

Tregs are T cells that suppress immunity and heavily infiltrate tumor tissues, regulating tumor proliferation, invasion, and metastasis. Metabolic reprogramming and microenvironmental changes generally play a role in promoting the function of Tregs: i) invasion of Tregs into tumor tissue by cytoskeletal rearrangement or secretion of chemokines; ii) induce of transcription factors or suppressive cytokines that promote the differentiation of T cells into Tregs; and iii) stimulation of the immunosuppressive function by regulating metabolism or promoting Treg secretion of functional cytokines. There are probably still chemokines, cytokines, and relevant signaling pathways to be discovered with the above modes of action. The effects of metabolism on Tregs are two-sided. Glycolysis can promote the proliferation of Tregs but it inhibits its immunosuppressive function. The metabolites of glycolysis can participate in other metabolic activities that promote Treg function. The effects of metabolic reprogramming on Tregs are not limited to the role of a single signaling pathway or a single type of metabolism. Instead, they are affected by changes in the microenvironment to regulate the cell function. The key transcription factor, HIF-1α, in the tumor microenvironment is also bidirectional in regulating Tregs. The expression of HIF-1α is increased under hypoxia. On one hand, it can inhibit the differentiation of Tregs by promoting glycolysis [119, 120], and on the other hand, it can promote the expression of Foxp3 and promote the differentiation of Tregs. The above phenomenon may be due to the different means of regulating the expression level of HIF-1α in cells. Different cytokines stimulates HIF-1α to activate different downstream signaling pathways, which leads to different functions of regulatory T cells [120]. Intracellular feedback regulation also affects the function of HIF-1α. Tregs affects its function according to the stimulation and regulation inside and outside the cell. In addition to traditional methods of regulation, non-coding RNAs can also affect the function of Tregs. miRs can be secreted by cells in exosomes, and then modulate the Tregs to act on the corresponding target regulatory cells. There are many kinds of non-coding RNAs, and their mode of action is complex. Further research is necessary to explore these regulatory mechanisms of Treg function. Many of the above experiments were performed in vitro, failing to completely simulate the complex dynamic balance of the tumor microenvironment, and Tregs are susceptible to this microenvironment. The experimental results may be inconsistent with the in vivo experiments; therefore, it is necessary to continue the exploration with rigorous experiments.

In addition to surgical treatment, chemotherapy, and radiotherapy, immunotherapy has gained increasing attention, including treatment of PD-L1 and its related signaling pathways [121], inhibitors of CTLA-4 [122], and CAR-T treatment [123]. Tregs as major immunosuppressive cells have an important influence on the occurrence and development of tumors. Although large numbers of Tregs are usually associated with poor clinical outcomes, the role of Tregs in colorectal cancer [124], head and neck squamous cell carcinoma [125], and esophageal cancer [49] remains controversial. This may be a result of the presence of chronic inflammation in tumor tissue that promotes abnormal cell proliferation, angiogenesis, and distant metastasis [126], whereas Tregs act as immunosuppressive cells that inhibit tumor development by reducing chronic inflammation. In addition, Tregs can also be divided into different subtypes according to the expression levels of Foxp3 and CD45RA, and the prognosis of patients with colorectal cancer is closely related to the infiltrating Treg subtype [127]. The complex role of Tregs in tumor progression may also be affected by the tumor microenvironment. Changes in tumor microenvironment and metabolic reprogramming can affect not only the differentiation of primary T cells into Tregs, but also its immune suppression function, and therefore also the balance of Tregs and Th17 by affecting the expression of Foxp3 and RORgt [128, 129]. The above factors comprehensively affect tumor progression. Therapeutic methods targeting Tregs are also receiving increasing attention. However, directly killing the Tregs in the tumor tissue does not improve the patient’s condition, because the adenosine produced by apoptotic Tregs will continue to inhibit the function of the effector cells [130]. Therefore, inhibiting the migration of Tregs to tumor tissues may have an effect on the treatment of cancer. Clinical trials have been conducted for treatment with chemokines, such as the use of monoclonal antibodies against CCR4 in the treatment of lymphoma, and a significant anti-tumor effect has been observed [131]. Regarding tumor infiltrating Tregs, Tregs can be depleted in tumor tissues by targeting the Treg surface-specific receptor, CD25, to inhibit tumor growth. Improving anti-CD25 antibodies and using them together with PD-1 antibodies may lead to a better therapeutic effect [132]. The fusion protein formed by IL-2 and diphtheria toxin can recognize IL-2R on the surface of Tregs to clear the cells. Treatment of renal cancer can effectively reduce the number of Tregs in the body, thereby achieving the purpose tumors treatments [133]. In addition to the expression of the transcription factor Foxp3, Tregs also expresses the transcription factor, Helios, to maintain stability. Deleting Helios will lead to the conversion of Tregs into effector T cells, which significantly delays tumor growth. Therefore, Helios targeting can be used as a feasible treatment strategy [134]. Similarly, targeting of Foxp3 can also help to regulate the expression of Tregs [135]. Moreover the treatment based on metabolic reprogramming has also been used in some immune diseases [136].

Based on the current status of immunotherapy, research still needs to be performed to improve our understanding of the mechanism of interaction between the tumor, Tregs and its subtypes, and other immune cells. Finding key cytokines, signaling pathways, non-coding RNAs that are relevant to Treg’ function is needed, and these will serve as potential targets for immunotherapy. The present review, which highlights the changes in the tumor microenvironment that result in changes in metabolites and thus affect the function of Tregs, enriches the theoretical basis of tumor immunosuppression, and may provide new directions for cancer immunotherapy.

## Abbreviations

AMPK: AMP kinase
CA: Carbonic anhydrase
CNS2: Non-coding sequence 2
CPT1A: Carnitine palmitoyltransferase-1A
CTLA: Cytotoxic T lymphocyte-associated antigen
FAO: Fatty acid oxidation
Foxp3: Forkhead/flanking helix nuclear transcription factor
GCK: Glucokinase
HIF: hypoxia-inducible factor
HREs: Hypoxia response elements
IDO: Indoleamine 2,3 dioxygenase
IL: Interleukin
LDH-A: Lactate dehydrogenase A
MCT: Monocarboxylate transporter
miRs: microRNAs
NFAT: Nuclear factor of activated T cells
OXPHOS: Oxidative phosphorylation
PD-1: Programmed cell death protein-1
PD-L1: Programmed death-ligand-1
RA: Retinoic acid
ROS: Reactive oxygen species
TGF: Transforming growth factor
TLR: Toll-like receptora
Tregs: Regulatory T cells
VEGF: Vascular endothelial growth factor
